# Shark fin ECG pattern in a patient with acute ascending aortic dissection

**DOI:** 10.1186/s12245-024-00732-z

**Published:** 2024-10-10

**Authors:** Alessio Caccioppola, Filippo Maria Russo, Enrico Molho, Lorenzo Fargione, Alessandro Guareschi, Federico Colombo, Alessandro Jachetti

**Affiliations:** 1 Department of Anesthesia and Critical Care, Fondazione IRCCS Ca’ Granda Ospedale Maggiore Policlinico, Milan, MI Italy; 2 Department of Clinical Sciences and Community Health, University of Milan, Milan, MI Italy; 3 Cardiology Department, Fondazione IRCCS Ca’ Granda Ospedale Maggiore Policlinico, Milan, MI Italy; 4 Emergency Department, Fondazione IRCCS Ca’ Granda Ospedale Maggiore Policlinico, Via F. Sforza 35, Milan, 20122 Italy

**Keywords:** Aortic dissection, Shark fin, ECG pattern - STEMI

## Abstract

The ‘shark-fin’ ECG-sign is a rare electrocardiographic pattern seen in ACS, typically associated with poor prognosis. However, its manifestation in acute aortic syndromes remains largely unexplored. Here, we present a case of a patient diagnosed with Ascending Aortic Dissection, who presented to ED with acute chest pain and shark-fin ECG-pattern.

**Supplementary Information:**

The online version contains supplementary material available at 10.1186/s12245-024-00732-z.

## History of presentation

**Fig. 1:**
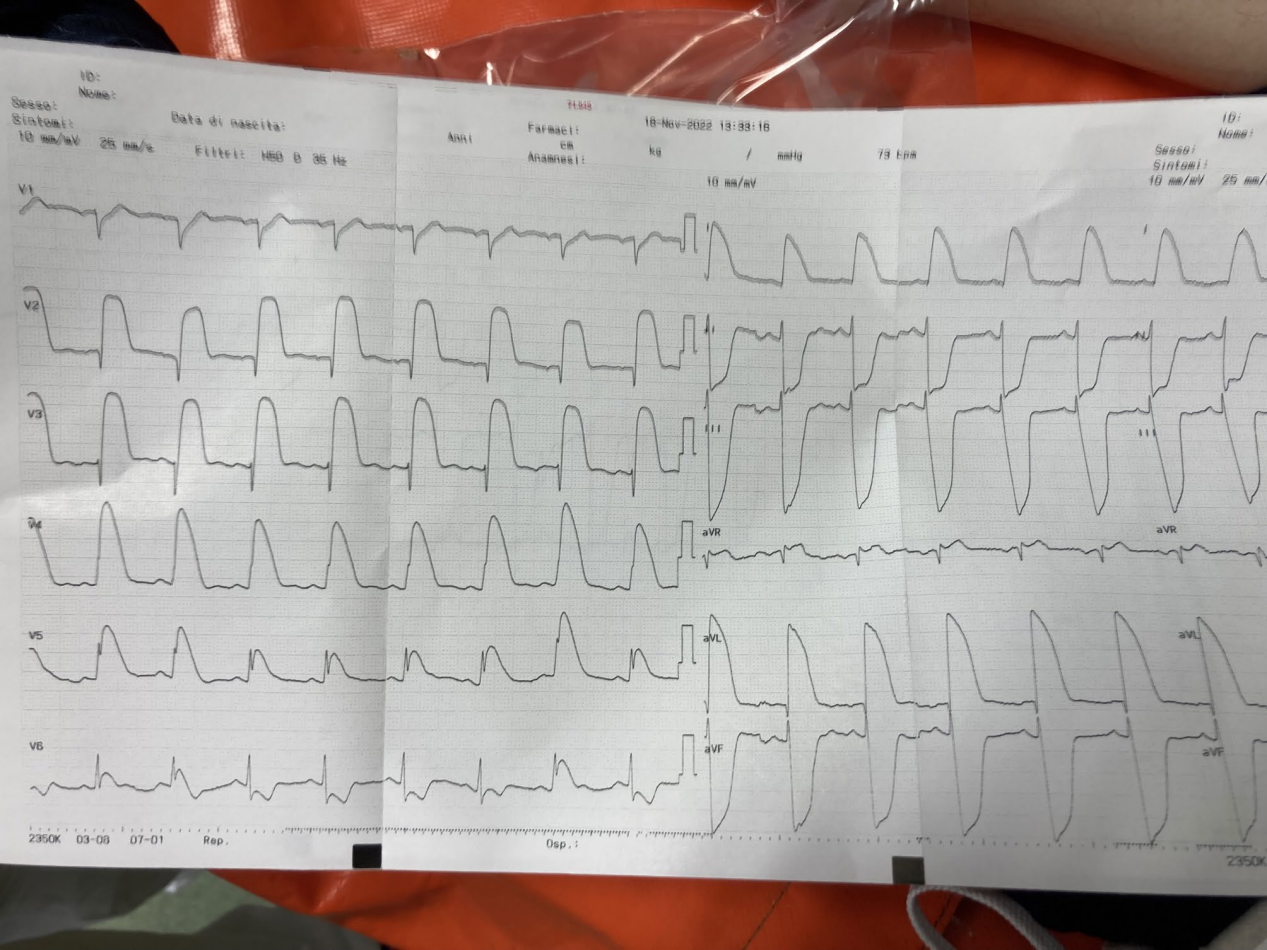
ECG at presentation in ED

A 59-year-old man with a history of hypertension and a previous transient ischemic attack presented to our ED with sudden-onset oppressive chest pain radiating to the left arm, accompanied by pre-syncope. Fortunately, the patient was already in the hospital main atrium when symptoms suddenly appeared and it was immediately referred to the Emergency Department. Upon initial evaluation, the patient appeared distressed, pale and diaphoretic with a blood pressure of 78/55 mmHg, so it was immediately transferred in the resuscitation area and the Cardiologist was paged. The patient complained a strong sternal oppressive pain with no radiation to the back, pulses were symmetric and regular, respiratory distress was not present with good peripheral oxygen saturation. No cardiac murmurs were detected during the physical examination. The ECG revealed a shark-fin pattern in the anterior leads (Fig. 1). A transthoracic echocardiogram was also performed but it was inconclusive due to a difficult acoustic window. Suspecting ST-elevation myocardial infarction (STEMI), the patient received an intravenous acetylsalicylic acid bolus of 250 mg and the cardiac catheterization laboratory was promptly activated for emergent percutaneous coronary intervention (PCI). Subsequent blood tests, received after the patient’s transfer to the catheterization laboratory, showed no significant elevation in cardiac troponin T, with only minimal increases in lactate dehydrogenase and creatine kinase levels, as expected due to the short presentation time from symptoms onset. The total time of permanence in the resuscitation room was 11 min, as registered from the ED’s IT-software.

## Differential diagnosis

In the presence of cardiac chest pain and an ECG consistent with the shark fin sign, an initial diagnosis of anterior ST-elevation myocardial infarction (STEMI) was considered more likely. However, given the coexistence of chest pain and hemodynamic instability, the Aortic Diseases European Guidelines [1] recommend a diagnostic algorithm focused on excluding acute aortic syndrome through additional investigations such as CT scan and transesophageal echocardiography. Nevertheless, in cases where ECG findings suggest a high-risk anterior STEMI, expedited referral to the catheterization laboratory is warranted without further investigations. It’s important to note that anterior ST-elevation myocardial infarction with extensive anterior ischemia can occur due to the extension of the dissection flap, which may occlude the left main coronary artery. Thus, ruling out aortic dissection requires additional parametric tests such as transesophageal echocardiography, performed in the catheterization laboratory, to assess for features such as a dilated aortic root, intimal flap, aortic regurgitation, or pericardial effusion. Differential diagnosis also involves distinguishing between coronary risk factors and clinical factors associated with a high pretest probability of acute aortic syndrome, such as chest pain of abrupt onset, systolic blood pressure difference, low diastolic blood pressure, or auscultation of a diastolic murmur of aortic regurgitation. Blood pressure measurements taken rapidly in both arms in the cardiac catheterization laboratory or the ED can help identify differences between the two sides, suggesting an immediate concern for type A aortic dissection. Furthermore, while tachycardia and hypotension may be related to myocardial infarction, they can also be indicative of aortic dissection with complications such as cardiac tamponade or aortic rupture.

A Tako-tsubo syndrome is another rare differential diagnosis to be considered in presence of an ECG Shark-fin pattern, as this association is described in literature [2, 3]. Anyway, in this particular case there were no clinical clues suggestive of this particular syndrome due to the severity and the rapid onset of the symptoms, so it was promptly ruled out.

Another rare condition associated with the Shark-fin ECG pattern is apical hypertrophic cardiomyopathy, as demonstrated by a recent case report by Tang et al. [4]. However, this diagnosis should be considered only after the more common differential diagnoses have been excluded.

### Investigations

The patient was urgently transferred to the Cath Lab in condition of hemodynamic instability. During the injection of contrast medium, suspicious images suggestive of a false lumen were evident at the level of the ascending aorta. Aortography was performed and revealed the presence of proximal aortic dissection with functional occlusion of the left main that was entirely perfused by the false lumen. Additionally, aortic insufficiency was observed with contrast regurgitation into the ventricle during aortography (See Supplementary Material Video 1_1, Video 1_2 e Video 1_3). Due to the evident clinical picture of type A aortic dissection, orotracheal intubation was performed and the patient was promptly transferred to the cardiac surgery operating room.

## Management

The patient underwent emergent surgery for a type A aortic dissection and extensive anterolateral infarction resulting from occlusion of the left coronary artery by the intimal flap. Given the patient’s unstable clinical condition, including cardiogenic shock and pulmonary edema, a preoperative CT scan was deemed impractical due to the high risk of sudden deterioration. Intraoperative transesophageal echocardiography (see Supplementary Material Video 2_1, Video 2_2, Video 2.3) revealed a severely reduced left ventricular ejection fraction (20–25%) and a type A aortic dissection originating from the sino-tubular junction at the level of the non-coronary cusp, extending throughout the aorta within the limits of exploration.

Ascending aorta replacement was performed under moderate hypothermic cardiac arrest (24 °C), with cardiopulmonary bypass (CPB) established through central arterial cannulation between the ascending aorta and the aortic arch using the Seldinger technique under echocardiographic guidance to ensure placement within the true lumen. Separation from CPB was impeded by severe biventricular dysfunction, necessitating initiation of full support with central Veno-Arterial ExtraCorporeal Membrane Oxygenation (VA-ECMO).

The postoperative course was complicated by multiple revision surgeries for bleeding. The patient was successfully weaned from ECMO on the 12th postoperative day but unfortunately succumbed to multiorgan failure five days later.

## Discussion

This case report illuminates the diagnostic and management challenges inherent in navigating the intersection of ST-segment elevation myocardial infarction (STEMI) and acute aortic dissection, particularly when confronted with the characteristic “shark fin” ECG pattern indicative of high-risk anterior STEMI [5].

In the realm of STEMI diagnosis, reliance on ECG findings serves as the cornerstone for prompt initiation of emergency reperfusion strategies, aligning with current Acute Coronary Syndrome-European Guidelines [6]. However, the presence of the shark fin sign on ECG signals a heightened risk of adverse cardiovascular events, including ventricular fibrillation and cardiogenic shock, with concomitant high in-hospital mortality rates, complicating the diagnostic landscape [5].

In our presented case, the initial interpretation of ST-segment elevation suggested a straightforward anterior myocardial infarction scenario. Yet, subsequent evaluation unveiled an underlying aortic dissection, wherein extension of the dissection flap led to left main coronary artery occlusion, mimicking acute atherothrombotic coronary occlusion. The potential ramifications of such a misdiagnosis are profound, as invasive interventions like cardiac catheterization hold the peril of exacerbating aortic dissection by inadvertently extending the flap within the aorta or coronary arteries through procedural maneuvers.

Furthermore, the discussion delves into the nuanced considerations surrounding peri-procedural drug therapy in the management of aortic dissection. While anticoagulants and antiplatelet agents are pivotal in STEMI management, their administration in the context of aortic dissection carries the peril of precipitating life-threatening bleeding complications. Moreover, the specter of cardiac tamponade, arising from retrograde dissection propagation and subsequent aortic wall rupture, underscores the imperative for judicious treatment stratification in these complex clinical scenarios.

## Conclusions

The shift from an initial diagnosis of anterior STEMI to the eventual recognition of aortic dissection underscores the critical necessity of broadening the differential diagnosis to encompass non-coronary etiologies of chest pain, especially when confronting hemodynamically unstable patients. Timely recognition and collaborative, multidisciplinary management play pivotal roles in optimizing patient outcomes, particularly in settings lacking on-site cardiac surgical capabilities. Thus, expedient transfer to specialized centers equipped with comprehensive resources is imperative for ensuring timely and effective intervention in such complex clinical scenarios. For this reason, when feasible, in the presence of clinical indications suggestive of acute aortic syndrome and ST elevations or a Shark-fin pattern on ECG, referral to a center equipped with both cardiac catheterization and cardiac/thoracic surgery capabilities should be considered.

## Learning objectives

While is widely diffuse the monoparametric diagnostic of STEMI with the 12 leads ECG, this case highlights the critical need for a multiparametric approach in assessing patients experiencing acute chest pain, not only in instances where ECG results are unclear, but also in cases with a strong suspicion of STEMI. Clinical awareness of non-coronary causes and pretest probability assessment are crucial in guiding diagnostic strategies. Transthoracic echocardiography, performed early in the evaluation process, can aid in differentiating between coronary and aortic pathologies, potentially preventing unnecessary interventions and optimizing patient outcomes. Additionally, recognizing features suggestive of acute aortic syndrome is crucial for expediting appropriate management strategies. In hemodynamically stable patients with a secured airway, consider invasive testing, such as transesophageal echocardiography (TEE) or a CT scan, to definitively rule out acute aortic syndrome.

## Electronic supplementary material

Below is the link to the electronic supplementary material.

Supplementary Material 1: Video 1_1. Cath-Lab aortography.

Supplementary Material 2: Video 1_2. Cath-Lab aortography.

Supplementary Material 3: Video 1_3. Cath-Lab aortography.

Supplementary Material 4: Video 2_1. Perioperative TEE SAX projection.

Supplementary Material 5: Video 2_2. Perioperative TEE 3CH projection.

Supplementary Material 6: Video 2_3. Perioperative TEE DISC2 projection.

## Data Availability

No datasets were generated or analysed during the current study.

## Abbreviations

ACS: Acute Coronary Syndrome
CPB: Cardiopulmonary Bypass
CT: Computer Tomography
ED: Emergency Department
IT: Information Technology
PCI: percutaneous coronary intervention
STEMI: ST-elevation myocardial infarction
VA-ECMO: Veno-Arterial ExtraCorporeal Membrane Oxygenation
